# Neonatal *Citrobacter koseri* Meningitis: Report of Four Cases

**DOI:** 10.1155/2014/195204

**Published:** 2014-02-19

**Authors:** Joana Rodrigues, Dalila Rocha, Fátima Santos, Anabela João

**Affiliations:** ^1^Pediatrics Department, Centro Hospitalar de Vila Nova de Gaia/Espinho, 4400-129 Vila Nova de Gaia, Portugal; ^2^Infancy and Adolescence Neurosciences Unit-Pediatrics Department, Centro Hospitalar de Vila Nova de Gaia/Espinho, 4400-129 Vila Nova de Gaia, Portugal; ^3^Neonatology Unit-Pediatrics Department, Centro Hospitalar de Vila Nova de Gaia/Espinho, 4400-129 Vila Nova de Gaia, Portugal

## Abstract

*Citrobacter koseri* is a rare cause of neonatal meningitis with predisposal for brain abscesses, and therefore responsible for high mortality and serious neurologic sequelae in this age group. We present the evolution and outcome of four cases of *C. koseri* meningitis. One of them developed brain abscesses and another one died. The cases show the bacteria's propensity for serious brain damage, despite early and adequate treatment, and the high risk of long-term neurologic complications in survivors, which imposes a close follow-up.

## 1. Introduction

*Citrobacter koseri *is a gram-negative bacillus which belongs to the family of Enterobacteriaceae. Although it is rare in the newborn, this bacterium has a strong neurotropism in this age group, causing meningitis and brain abscesses. For this reason, it is associated with high rates of mortality, morbidity, and long-term sequelae [1].

This propensity for *C. koseri *to damage the central nervous system is still poorly understood. Experimental studies suggest a specific outer membrane protein as the likely neurovirulence factor [2].

Former reports of *C. koseri *meningitis have been published, most of them associated with neurological complications, including brain abscesses. The authors present the analysis of four cases of *C. koseri *meningitis, which occurred in a neonatal intensive care unit of a tertiary care hospital, over ten years, emphasizing its clinical acute and long-term neurological complications and comparing them with the published literature on this subject.

## 2. Case Reports

*Case 1*. A male newborn was born after a 39-week uncomplicated pregnancy by cesarean delivery, with Apgar score 6/8/8 and birth weight 3420 g. He was admitted to the neonatal intensive care unit (NICU) at birth with respiratory distress. By that time he had a normal chest radiograph, white blood count (WBC) and C reactive protein (CRP). He had a favorable outcome without ventilatory support and was discharged at 48 hours of life.

He was taken to the emergency department on the sixth day of life with fever, poor feeding, irritability, hypotonia, and poor perfusion. The WBC revealed leukocytosis with neutrophilia and CRP was 2.6 mg/dL. The cerebral spinal fluid (CSF) analysis showed pleocytosis and hypoglycorrachia. Intravenous antibiotic therapy with ampicillin, gentamycin, and cefotaxime was started immediately after collecting culture samples. Still on the first day of admission he had an episode of generalized tonic-clonic seizure solved with a single dose of phenobarbital. Transfontanellar ultrasound (TFUS) performed on the second day of disease revealed right frontal echogenicity suggestive of infarction/ischemia. On the fourth day the initial blood culture was negative and CSF culture revealed a *C. koseri *sensitive to gentamycin and cefotaxime and resistant to ampicillin. CSF culture was repeated on the seventh day and the result was negative. On the eighth day of hospitalization, the brain magnetic resonance imaging (MRI) described bilateral frontal encapsulated lesions with haemorrhagic component following meningovasculitis.

Serial ultrasounds showed no evolution until the twenty first day, when a large porencephalic cyst was seen on the location of the previous changes.

He was evaluated by an ophthalmology specialist by the thirty fifth day because of a new acute fixed strabismus. By that time, brain MRI was repeated, describing lesions compatible with probable brain abscesses. The case was discussed with neurosurgery, having been decided that there was no indication for drainage. The clinical outcome was favorable, except for the strabismus, which became bilateral. Six weeks of antibiotics were completed. He was discharged with no other changes on the neurological examination.

About 2 weeks after completing treatment the brain MRI revealed resolution of the haemorrhagic lesions with bilateral frontal encephaloclastic lesions.

This patient was followed in neurodevelopment consultation. At 3 months of life he presented an adequate development and normal neurological examination. At 6 months he was hospitalized with West syndrome. The electroencephalogram showed hypsarrhythmia intercepted by multifocal and generalized paroxysmal discharges and the brain MRI revealed a large right frontal cystic lesion communicating with the right lateral ventricle with no mass effect. Antiepileptic therapy was started but throughout the years he kept a difficult control epilepsy with the need of frequent treatment adjustments. He is now under medication with valproic acid.

At preschool age this boy revealed developmental delay in fine motricity and language skills and he initiated developmental therapies. The brain MRI showed a voluminous porencephalic cyst with similar aspects.

At 8 years old his epilepsy was controlled and by that time he was attending regular school without learning disabilities. Today he is 9 years old.

*Case 2*. A twenty-seven-week-old male newborn was born by cesarean delivery 3 days after membrane rupture and maternal fever, with an Apgar score 2/8/8 and birth weight 1070 g. He was immediately admitted to the NICU and treated with surfactant and mechanic ventilation (invasive during 7 days and then noninvasive). At birth, WBC and CRP were normal. Blood culture was sterile. He fulfilled ten days of treatment with ampicillin and gentamycin.

On the twenty seventh day of life he started recurrent apnea and bradycardia. CRP was 9.16 mg/dL and CSF analysis revealed no pleocytosis, hyperproteinorrachia, and hypoglycorrachia. Antibiotherapy was started with vancomycin and amikacin. Blood and CSF culture showed *C. koseri *resistant to ampicillin. Vancomycin was changed to meropenem. At 48 h after starting antibiotics the CSF culture was sterile.

This newborn had a favorable clinical evolution. TFUS were persistently normal. He completed 21 days of antibiotherapy. At discharge, he had a normal neurological examination.

At 5 months old (chronological age) peripheral spasticity of the four limbs was detected. Brain MRI at 8 months revealed a delay of mielinization related to prematurity. He developed a progressive tetrapyramidal syndrome and development delay. At 5 years old he had spastic diplegia, language delay and attention deficit hyperactivity disorder. At 8 years old, learning disabilities were found. Today the boy is 9 years old.

*Case 3*. Twin monoamniotic monochorionic pregnancy complicated by feto-fetal transfusion was diagnosed at 26 gestational weeks. Amnioreductions were started with probable iatrogenic amniotomy leading to pregnancy interruption at 29 weeks.

Female newborn was delivered by cesarean section with Apgar score 5/7/9 and birth weight 1300 g. She was admitted to the NICU, needing noninvasive ventilation and partial exchange transfusion. She was clinically stable up to 48 h of life, when she developed frequent apnea. WBC revealed leucopenia and thrombocytopenia and lumbar puncture was unsuccessful. Vancomycin, cefotaxime, and amikacin were instituted. By 72 h of life deterioration occurred, with poor peripheral perfusion, cardiac murmur suggestive of persistent ductus arteriosus, and worsening of thrombocytopenia requiring platelet transfusion. Invasive ventilation was initiated. TFUS revealed a right frontoparietal hyperechogenicity. Progressive clinical and analytic impairment occurred, with haemodynamic instability, anuric hyperkalemic renal insufficiency, and metabolic acidosis. Treatment was started with amines, diuretics, insulin, ion exchange resin, and bicarbonate. On the sixth day of life she developed an epileptic status with the need of treatment with phenobarbital, midazolam, and phenytoin. On the seventh day TFUS was repeated and showed global right hemisphere and left occipital hyperechogenicity with lenticulostriate vasculopathy and undefined brain structures. She died on the eighth day of life. *C. koseri* resistant only to ampicillin was found in blood culture, urine culture, central venous catheter, and ocular exudate.

*Case 4*. The case was a female newborn, from a twin monochorionic diamniotic gestation. Fetal death of the other twin was diagnosed at 34 gestational weeks leading to pregnancy interruption.

She was born by cesarean section with Apgar score 7/10/10 and birth weight 2180 g. She was admitted to the NICU, remaining clinically stable until the eighth day of life, when she began subfebrile temperature, grunting, irritability, and hyperglycemia. CRP was 1.89 mg/dL and CSF analysis revealed pleocytosis. Antibiotic therapy was started with ampicillin, amikacin, and cefotaxime. She showed slow but progressive clinical improvement. On the tenth day of life the TFUS revealed a left frontal and prefrontal hyperechogenic zone.

On the thirteenth day of life blood and CSF culture showed *C. koseri* resistant to ampicillin and this antibiotic was stopped. CSF culture came sterile after 48 h of antibiotics.

**Figure 1:**
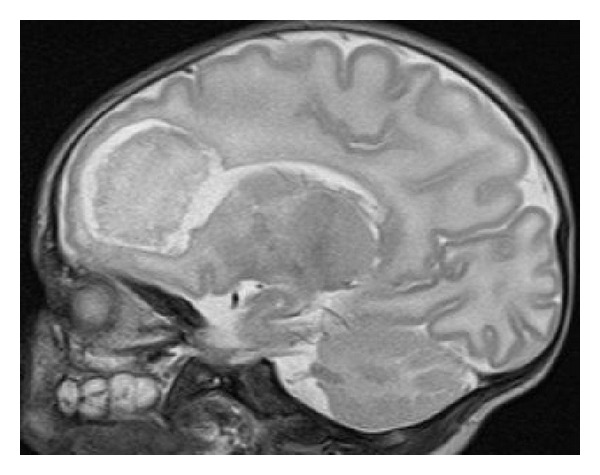
Cystic encephalomalacia involving the bilateral frontal periventricular white matter, reaching the left globus pallidus and caudate nucleus.

On the ninth day of life (eleventh day of disease) brain MRI showed frontal cystic encephalomalacia reaching the basal ganglia (Figure 1). Antibiotherapy was kept for 21 days. At discharge the newborn had a normal neurological examination.

At 6 months old (chronological age), she developed spasticity of the lower limbs and started physiotherapy. Today she is 8 months old.

## 3. Discussion

Species of the genus *Citrobacter *are responsible for neonatal sepsis and meningitis in sporadic (including through vertical transmission) or epidemic (if within nosocomial outbreaks) forms. Doran, in 1999, described 6 vertical transmission cases and 12 cases of nosocomial infection in a universe of 51 cases of meningitis caused by *Citrobacter*. The remaining were named sporadic once the bacteria's origin was not found [1].

In none of our cases was it possible to establish the occurrence of vertical or nosocomial transmission.

In our four cases the clinical suspicion of sepsis with or without meningitis was corroborated by both analytical markers of infection and subsequent identification of the microorganism in blood and CSF culture (except in case 3, as it was not possible to collect CSF samples).

*C. koseri *is often associated with brain abscesses. It is unclear in the published literature how often sepsis occurs without meningitis or meningitis occurs without abscesses, but in that same review by Doran, 68.6% of cases of meningitis caused by *Citrobacter* were complicated with brain abscesses [1]. On the other hand, most recently published clinical cases often describe abscesses approach and evolution [3–7].

Only one of our cases did not show any brain changes during the acute phase of the disease. In the other cases there was frontal brain involvement.

In all of the cases antibiotherapy was started within the first hours of evolution of the disease and included at least one antibiotic to which the bacteria was sensitive. Even so, it did not prevent the progression to brain disease in most cases. As described in literature [1, 3, 4, 8, 9], all the isolated *C. koseri *were resistant to ampicillin and were treated with combination antibiotherapy following the clinical evolution and antibiogram (usually an aminoglycoside and a 3rd generation cephalosporin or carbapenem).

In terms of treatment duration, it is suggested in the literature that treatment of meningitis is at least 21 days and in the case of brain abscesses 4 to 6 weeks and eventual drainage [1, 7, 8, 10]. All our patients completed 21 days of treatment, except for the patient with brain abscesses whose treatment was longer (6 weeks) and whose discussion with neurosurgery took to the decision not to perform drainage.

As mentioned above, *C. koseri *meningitis is associated with high rates of mortality and morbidity: 30% of cases are fatal and 50% of survivors are left with neurologic sequelae [1], compared with 15.9% mortality and 21.6% sequelae of neonatal meningitis in a review published in 2013 by Hamounda et al. [11]. Among the most frequent sequelae are mental retardation and epilepsy.

In our group of cases, one of the patients had a fatal outcome. The other cases ended up evolving favorably in the acute phase of illness. However, afterwards they developed neurologic symptoms.

The patient from case 1 developed significant sequelae chargeable to the infection. The imaging changes conditioned epilepsy and delayed psychomotor development.

Regarding the fourth case, despite the favorable outcome in the acute phase of the disease, the spasticity observed after 6 months of life is already evidence of sequelae conditioned by cystic encephalomalacia.

The child on case 2 also developed long-term neurologic changes, but those changes along with the lack of significant findings in the TFUS and brain's MRI suggest a stronger relation with the extremely preterm age of birth.

This group of cases shows the possible and frequent devastating effects of the infection by *C. koseri *in the newborn, confirming its propensity to affect the meninges and brain. Our cases also show a predilection for frontal injury which has not been described in literature yet.

The clinical course is usually more aggressive than in meningitis by other bacteria and the prevalence of brain involvement is high, even when early and adequate antibiotherapy is performed. Clinical and imagiological (TFUS) monitoring is very important in order to early detect and treat complications.

In survivors, the high risk of long-term neurologic sequelae imposes a close follow-up.
